# *In Silico* Insights into Protein-Protein Interactions and Folding Dynamics of the Saposin-Like Domain of *Solanum tuberosum* Aspartic Protease

**DOI:** 10.1371/journal.pone.0104315

**Published:** 2014-09-04

**Authors:** Dref C. De Moura, Brian C. Bryksa, Rickey Y. Yada

**Affiliations:** 1 Biophysics Interdepartmental Group, University of Guelph, Guelph, Ontario, Canada; 2 Department of Food Science, University of Guelph, Guelph, Ontario, Canada; Russian Academy of Sciences, Institute for Biological Instrumentation, Russian Federation

## Abstract

The plant-specific insert is an approximately 100-residue domain found exclusively within the C-terminal lobe of some plant aspartic proteases. Structurally, this domain is a member of the saposin-like protein family, and is involved in plant pathogen defense as well as vacuolar targeting of the parent protease molecule. Similar to other members of the saposin-like protein family, most notably saposins A and C, the recently resolved crystal structure of potato (*Solanum tuberosum*) plant-specific insert has been shown to exist in a substrate-bound open conformation in which the plant-specific insert oligomerizes to form homodimers. In addition to the open structure, a closed conformation also exists having the classic saposin fold of the saposin-like protein family as observed in the crystal structure of barley (*Hordeum vulgare* L.) plant-specific insert. In the present study, the mechanisms of tertiary and quaternary conformation changes of potato plant-specific insert were investigated *in silico* as a function of pH. Umbrella sampling and determination of the free energy change of dissociation of the plant-specific insert homodimer revealed that increasing the pH of the system to near physiological levels reduced the free energy barrier to dissociation. Furthermore, principal component analysis was used to characterize conformational changes at both acidic and neutral pH. The results indicated that the plant-specific insert may adopt a tertiary structure similar to the characteristic saposin fold and suggest a potential new structural motif among saposin-like proteins. To our knowledge, this acidified PSI structure presents the first example of an alternative saposin-fold motif for any member of the large and diverse SAPLIP family.

## Introduction

Pepsin-like aspartic proteases (APs) constitute a family of endopeptidases found in all kingdoms of life [1]. APs of plant origin are generally homologous to members of the A1 family of APs (http://merops.sanger.ac.uk) [2] sharing similar primary and bilobal tertiary structures wherein two lobes are separated by a large active site cleft containing two catalytic aspartic acid residues, and low pH optima. However, plant APs are unique in that they frequently contain an extra 100-residue domain inserted in the C-terminal lobe, distinguishing them from their microbial and animal counterparts [3]. This extra domain, termed the plant-specific insert (PSI) or plant-specific sequence (PSS) [4]–[7], belongs to the saposin-like protein (SAPLIP) family and contains the Saposin B (Sap B) protein domain architecture [8]. Physiologically, SAPLIPs exhibit varied functionalities manifested primarily in their abilities to target, bind and/or perturb membranes, sometimes involving the ability to permeabilize and/or induce vesicle fusion [8]–[11]. Examples of SAPLIP function include sphingolipid degradation and antigen presentation [12], haemolytic activity (*Na*-SLP-1 and *Ac*-SLP-1) [13], antimicrobial and cytolytic activity (NK-lysin and granulysin) [14], [15] and fusion of large unilamellar anionic vesicles *in vitro* (Sap C and recombinant PSI expressed without the parent AP) [9]–[11]. Recombinantly expressed free-form potato PSI has been shown to display potentially useful functionalities *in vitro* including antimicrobial activity against both plant and human pathogens [16], as well as anticancer activity against leukaemia cells without having lymphocyte toxicity [17].

Although SAPLIPs share low sequence identity and exhibit a multitude of functions in a variety of organisms, there have only been two discrete SAPLIP conformations observed to date. With the exception of granulysin [18], all known SAPLIPs have a characteristic pattern of 6 cysteines that form 3 disulfide bridges. The predominant tertiary structure is a substrate-free closed form first elucidated by NMR structure determination of porcine NK-lysin, and subsequently observed for all known SAPLIPs (see References [13], [14], [18]–[22]). This closed form, the classic saposin fold, is distinguished by a compact globular structure consisting of a 4 or 5 α-helix distorted bundle packed into an oblate spheroid.

By contrast, a second SAPLIP structural variant exists having an extended open conformation, first observed for Sap C bound to SDS micelles, that resembles two side-by-side boomerangs [19]. Unlike the compact structure seen in closed SAPLIPs, lipid-bound Sap C opens in a jackknife-like fashion thereby exposing the normally buried hydrophobic core to accommodate lipid interactions [23]. This V-shaped configuration has also been shown in Sap A bound to various amphiphiles [24]. The V-shaped SAPLIP configuration is also observed in Sap C homodimers in the absence of bound lipids [22]. As with Sap C bound to SDS micelles and Sap A lipoprotein discs, ligand-free Sap C jackknifes open at hinge points at the helix-helix junctions between the first two and last two helices. These hinge points allow Sap C monomers to open up and adopt an extended V-shape configuration forming domain-swapped homodimers. Hydrophobic regions are thus sequestered from their aqueous environment as the two interfaces come together yielding a hydrophobic core within the dimer. This jackknife opening mechanism serves to demonstrate the conformational flexibility of some SAPLIPs afforded by the helix-helix junctions. The ability to open and close allows for both membrane interactions and oligomerization [20], [22], [24].

Only two PSI structures (PDB IDs 1QDM and 3RFI), both resolved by X-ray crystallography, have been elucidated thus far: the inactive precursor structure of barley (*Hordeum vulgare* L.) phytepsin (HvAP) [25] and recombinant PSI of *Solanum tuberosum* (potato) AP [11]. Barley PSI was shown to have the archetypical compact saposin fold wherein the N- and C-termini remained attached to the C-terminal lobe of its parent phytepsin. By contrast, free form potato PSI adopts the less commonly observed open conformation as a homodimer analogous to that of ligand-free Sap C. Like their SAPLIP homologues, PSIs have been shown to induce vesicle leakage and fusion as well as having roles in plant vacuolar targeting [3], [11], [26], [27].

To gain insight into the structural determinants of the PSI’s pH-dependence for activity, the present study sought to elucidate and compare the protein dynamics and structural characteristics of the PSI in active and inactive pH conditions. Furthermore, the folding dynamics of the PSI open extended SAPLIP structure were investigated to clarify how the PSI tertiary structure relates to the typical closed SAPLIP fold.

## Results

### The PSI dimer forms a stable complex regardless of pH

**Figure 1:**
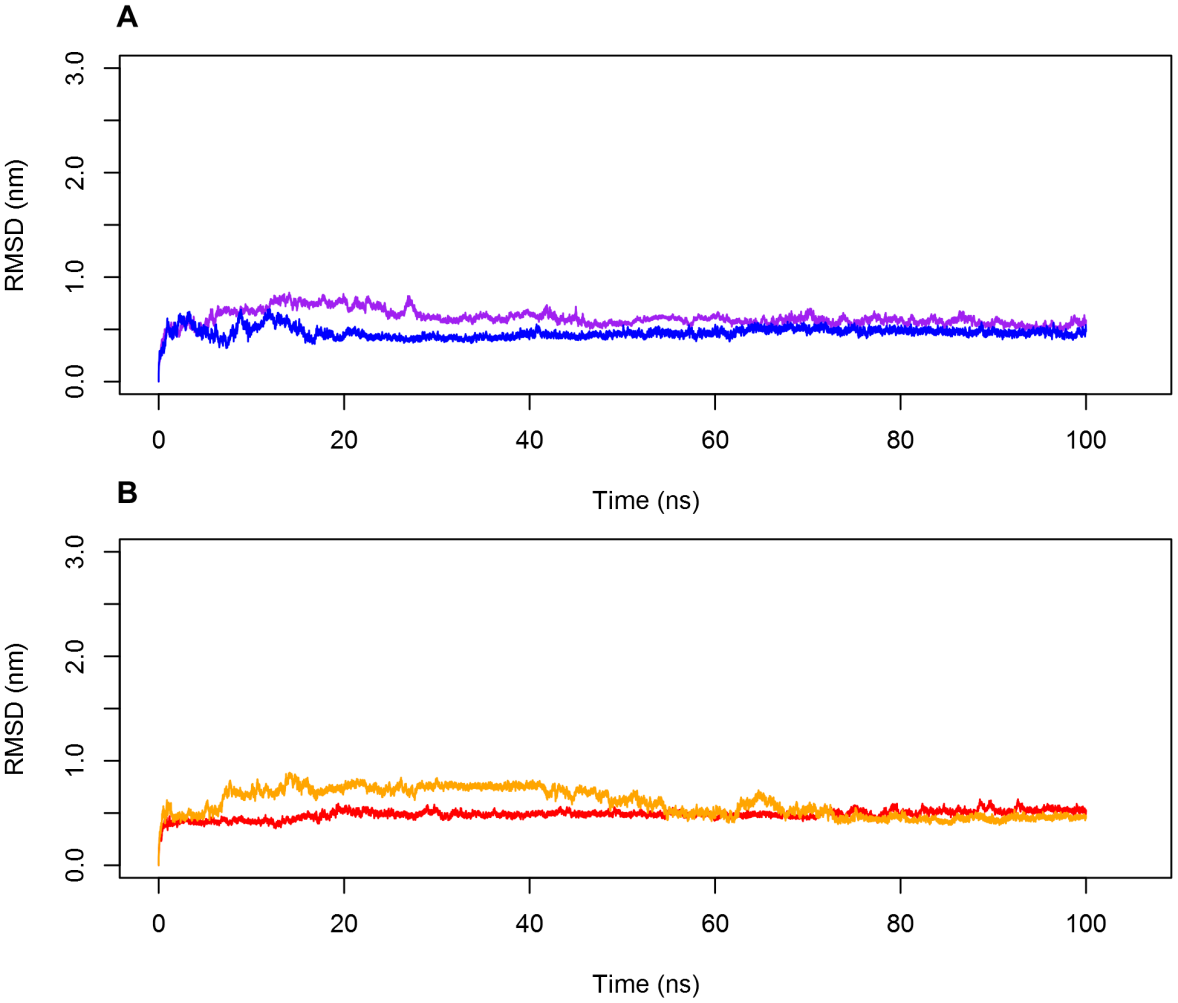
The backbone RMSD of the PSI dimer as a function of time. Backbone root-mean-square deviation (RMSD) of the PSI dimer at pH 3.0 (**A**) and pH 7.4 (**B**) indicated little deviation, evidenced by the low RMSD of the PSI backbone atoms for both peptides comprising the PSI homodimer. Colours identify the individual peptide chains within the dimer.

**Figure 2:**
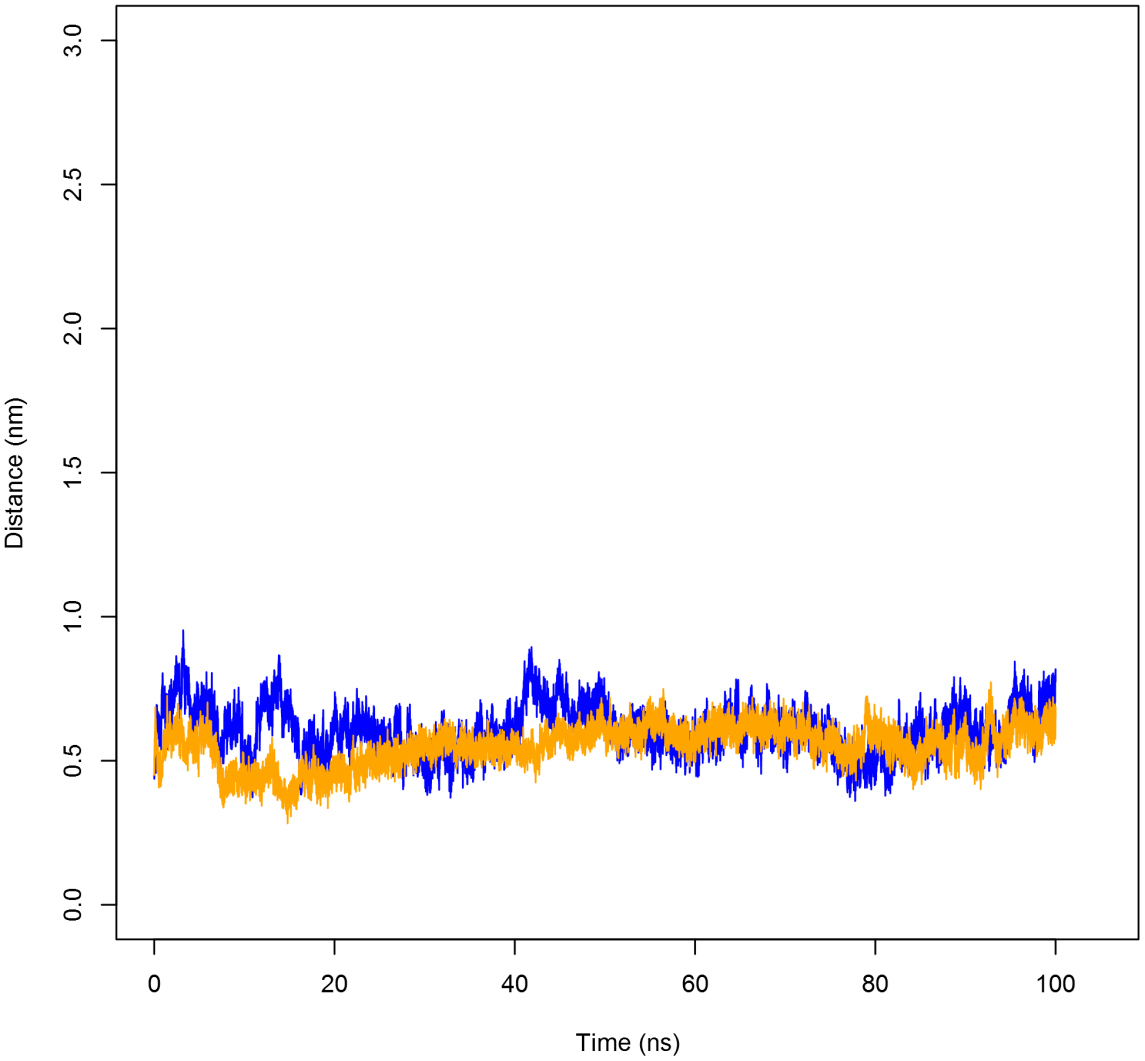
Distance between monomers of the PSI homodimer measured at the centres of mass (COMs) of each peptide. Distance between the two monomers of the PSI dimer revealed that the peptides maintained steady contact at the dimer interface regardless of pH. Both pH 3.0 (blue line) and pH 7.4 (orange line) simulations maintained an average distance of approximately 6 Å throughout the trajectories.

**Figure 3:**
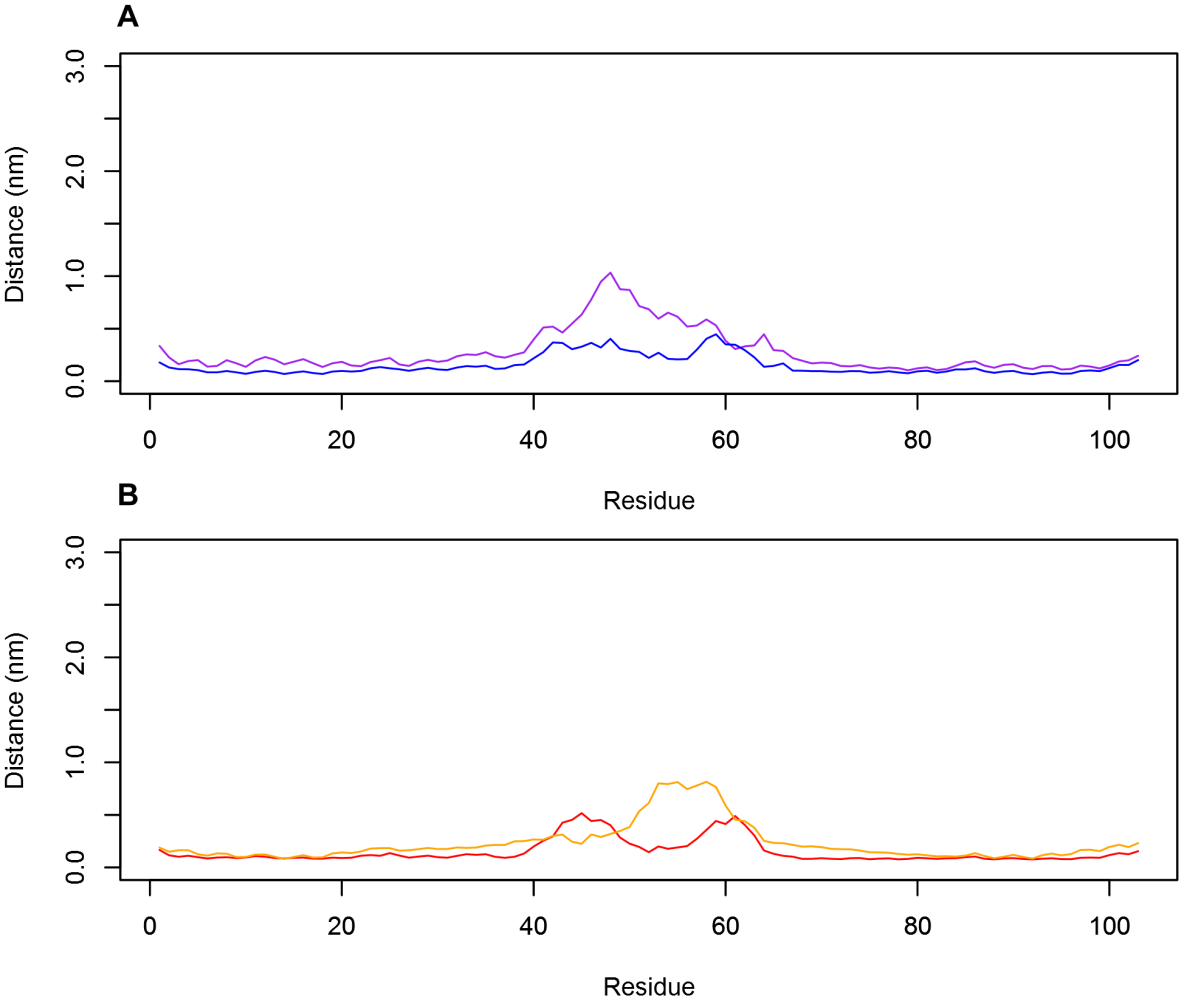
The C_α_ root-mean-square fluctuations (RMSFs) of the PSI dimer as a function of time. The C_α_ RMSFs for helices at pH 3.0 (**A**) and pH 7.4 (**B**) were consistent throughout the time course of the simulations, remaining below 5 Å for the helical regions. Fluctuations of up to 10 Å were noted for the flexible linker region, in agreement with the hypothesis that the linker region is intrinsically disordered, providing evidence of PSI dimer secondary structure stability regardless of pH. Colours identify the individual peptide chains within the dimer.

Equilibrium molecular dynamics simulations of the dimer complex in acidic (pH 3.0) and neutral (pH 7.4) conditions revealed that the PSI dimer is stable regardless of pH, evidenced by low and relatively constant root-mean-square deviation (RMSD) values of the dimer trajectories when fitted to the initial coordinates of the crystal structure (Figure 1). As measured at the centre of mass (COM) of the individual monomers within the dimer complex, each monomer maintained steady contact at the dimer interface throughout the time course of each simulation (Figure 2). Further examination of the trajectories showed little fluctuation in the residues comprising helical regions. The C_α_ root-mean-square fluctuation (RMSF) for helices remained consistent through 100 ns and deviated little from the crystal structure (PDB ID 3RFI) providing further evidence of dimer stability (Figure 3).

### Influence of pH on PSI dimer dissociation

Analogous to AFM pulling [28] and optical tweezers experiments [29], steered molecular dynamics (SMD) [30] simulations can be used to direct behaviour within a reduced number of degrees of freedom towards a particular state or phenomenon of interest. The efficiency of this technique can be exploited to study phenomena not normally accessible by conventional timescales due to the computational expense of traditional MD simulations. SMD simulations typically use pulling velocities that are orders of magnitude higher than those used in AFM pulling or optical tweezers experiments, resulting in comparatively higher pulling forces. SMD is useful in exploring underlying processes involved in the dissociation of a dimer complex as evidenced in previous studies on unbinding pathways of proteins and substrates [31]–[35].

As a function of the distance between two molecules, the 1D potential of mean force (PMF) along a desired reaction coordinate (ξ) can be calculated [36], [37]. In particular, it is the ability of the PMF, or free energy, to quantitatively describe Δ*G*_dissociation_ of a protein-ligand or dimer complex of interest. Although a number of ways of determining PMF exist [38], [39], the umbrella sampling method was chosen for its efficient sampling along the reaction coordinate [40]. Using the umbrella sampling method in the context of dimer dissociation, an umbrella biasing potential was applied to restrain one monomer at increasing distances from the second reference monomer as measured between the respective centres of mass. As opposed to conventional MD simulations, the use of the restraining potential allowed for increased sampling of conformational space at defined positions along the reaction coordinate, resulting in a series of biased histograms. The weighted histogram analysis method (WHAM) was then used to combine the individual distributions and extract the unbiased PMF in a manner similar to [41].

**Figure 4:**
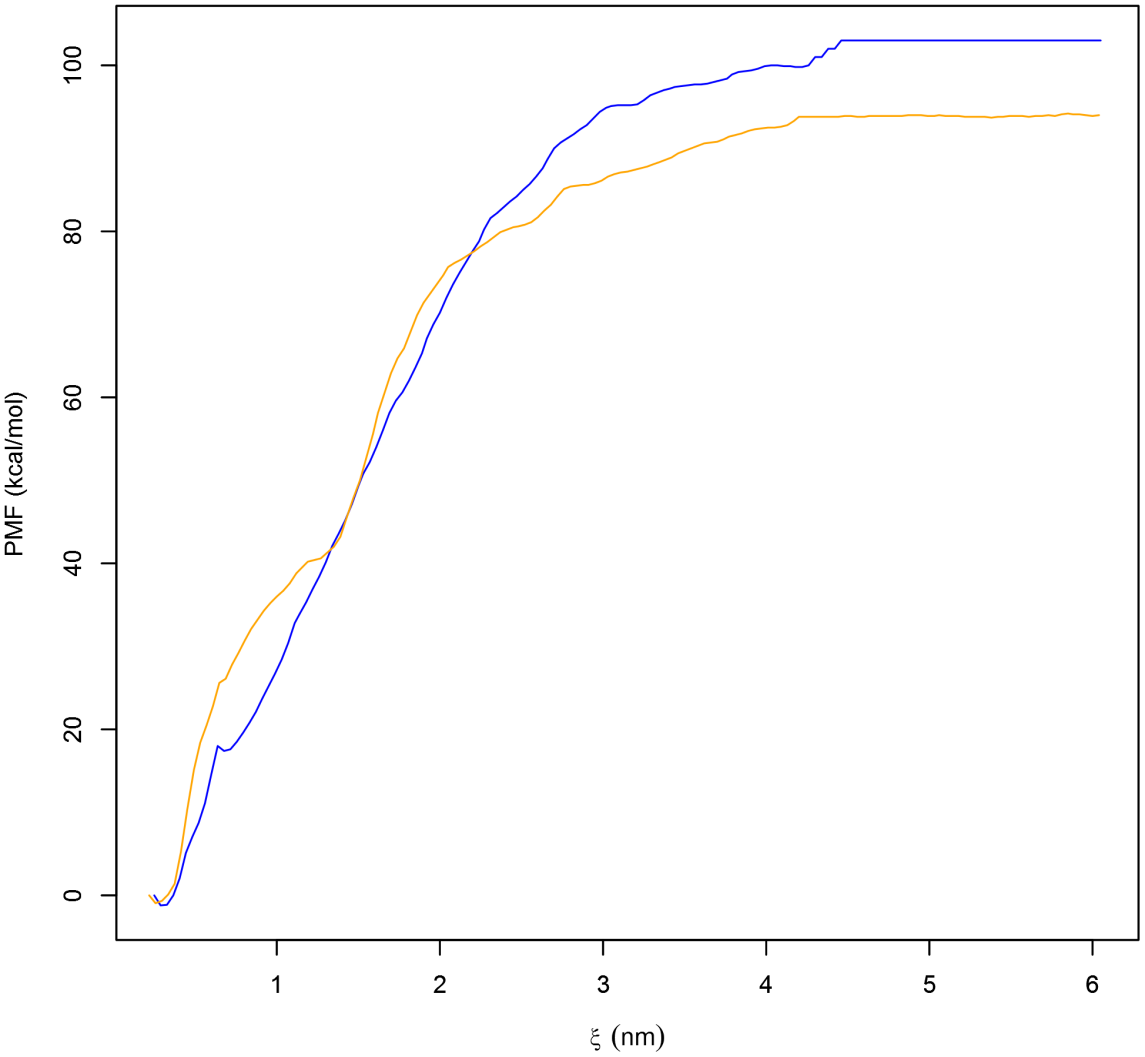
The potential of mean force (PMF) as a function of the distance between the COMs of PSI monomers. The PMF as a function of the intra-peptide distance between the PSI dimer monomers at pH 3.0 (blue line) and pH 7.4 (orange line) revealed that dissociation of the dimer requires increased energy as pH is lowered from pH 7.4 (95.7 kJ mol^−1^) to pH 3.0 (108.8 kJ mol^−1^), possibly the result of charge neutralization of carboxylate groups at acidic pH thereby minimizing charge-charge repulsion.

To assess the potential influence of pH on the dissociation of the PSI dimer and gain insight into the unbinding mechanisms of the dimer complex, SMD simulations were performed in combination with umbrella sampling and WHAM. Equilibrium MD simulation structures were used as starting configurations for SMD, and pulling simulations were performed for acidic (active; pH 3.0) and neutral (inactive; pH 7.4) conditions in which one monomer (chain B) was pulled away from an immobile reference peptide (chain A) along the z-axis such that the distance between their centres of mass increased as the two peptides were pulled apart from one another. Although the PSI is optimally active at pH 4.5, pH 3.0 was used here since the pH 4.5 dimer is not sufficiently soluble to conduct ongoing monomer-dimer equilibrium experiments whose preliminary data indicate that PSI exists as a dimer under acidic conditions (unpublished data). The resultant trajectories were then used to generate the windows for umbrella sampling, and WHAM was used to extract PMF associated with dimer dissociation (Figure 4). The PMF profiles indicated that a significant amount of free energy was required to instigate dissociation with Δ*G*_dissociation_ values of 108.8 kJ mol^−1^ at acidic pH and 95.7 kJ mol^−1^ at neutral pH. The high free energy barrier to dissociation was suggestive of strong intermolecular protein-protein interactions. This was expected and reasonable as the probability of water contacting the hydrophobic undersides of the respective PSI monomers is minimized by maintaining strong contacts at the dimer interface [42], thereby sequestering hydrophobic residues from solvent and thus stabilizing dimer quaternary structure.

While the PMF profiles describing both the acidic and neutral pH dimer dissociations were similar, it should be noted that the Δ*G*_dissociation_ for the PSI at pH 3.0 was almost 13% larger than at neutral pH. Although the two monomers maintained similar contact distances throughout the time course of the simulation, the difference in Δ*G*_dissociation_ (Figure 2) may be explained by differences in ability to preserve contact at the hydrophobic interfaces. Compared to the pH 7.4 simulation in which all residues were in their standard state, histidine as well as all glutamic and aspartic acid residues were protonated in the acid simulation resulting in charge neutralization and mitigation of electrostatic repulsion among the expansive number of negatively charged residues. This would result in the stabilization of the dimer as movement of the two monomers away from each other would be restricted by the dominant hydrophobic interactions at the dimer interface and the higher free energy requirement for dissociation.

### The closed saposin-fold conformation is the dominant structure adopted by monomeric PSI

**Figure 5:**
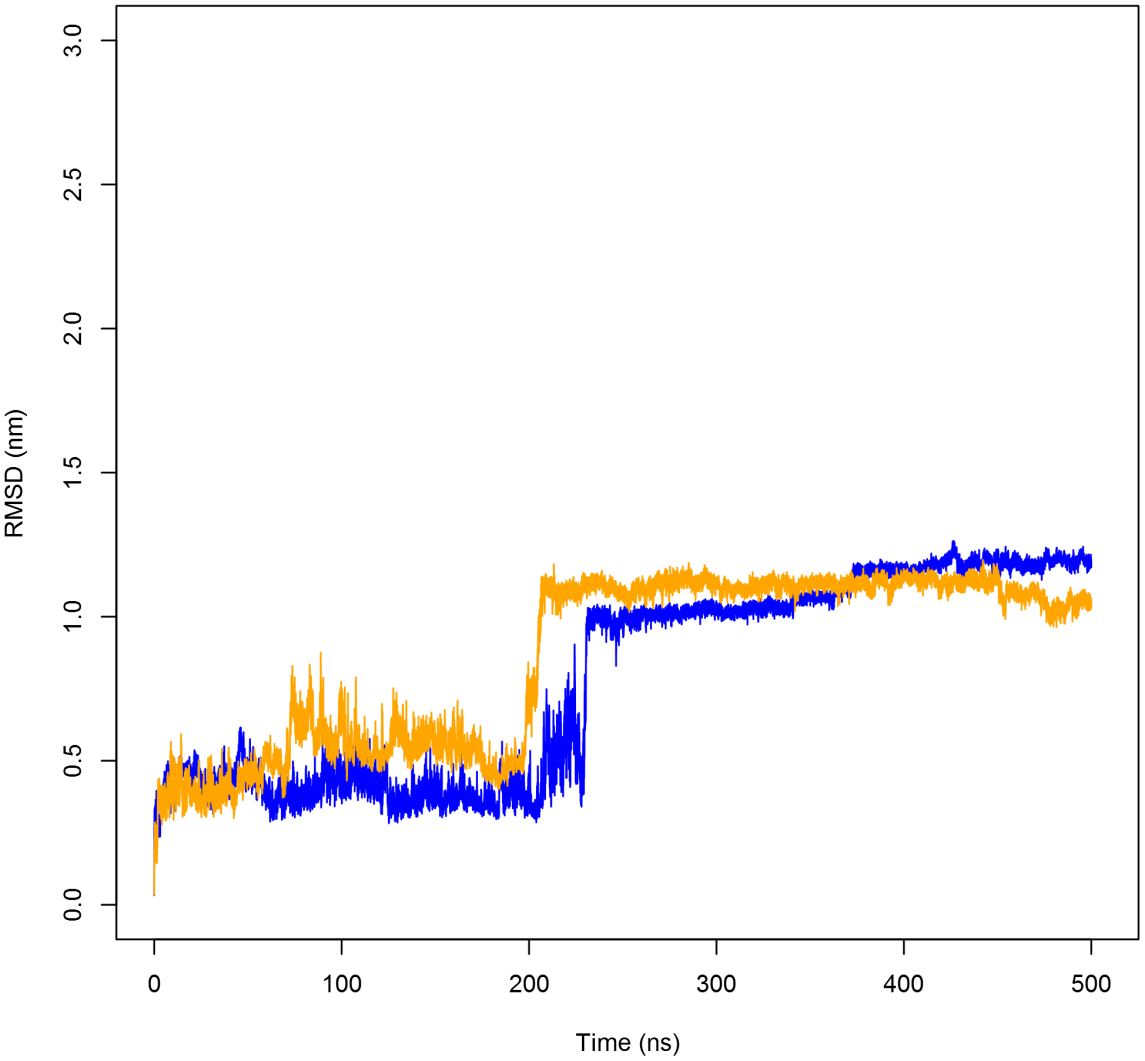
The backbone RMSD of PSI monomers as a function of time. Backbone RMSD of the PSI monomer at pH 4.5 (blue line) and pH 7.4 (orange line) are presented. The PSI monomer maintains its overall tertiary structure at both pH 4.5 and pH 7.4 similar to that of the native dimer structure until an abrupt change in RMSD at 230 ns and 198 ns for pH 4.5 and pH 7.4, respectively. At these times, the PSI jackknifes closed and adopts saposin-like fold characteristic of all known SAPLIP members.

**Figure 6:**
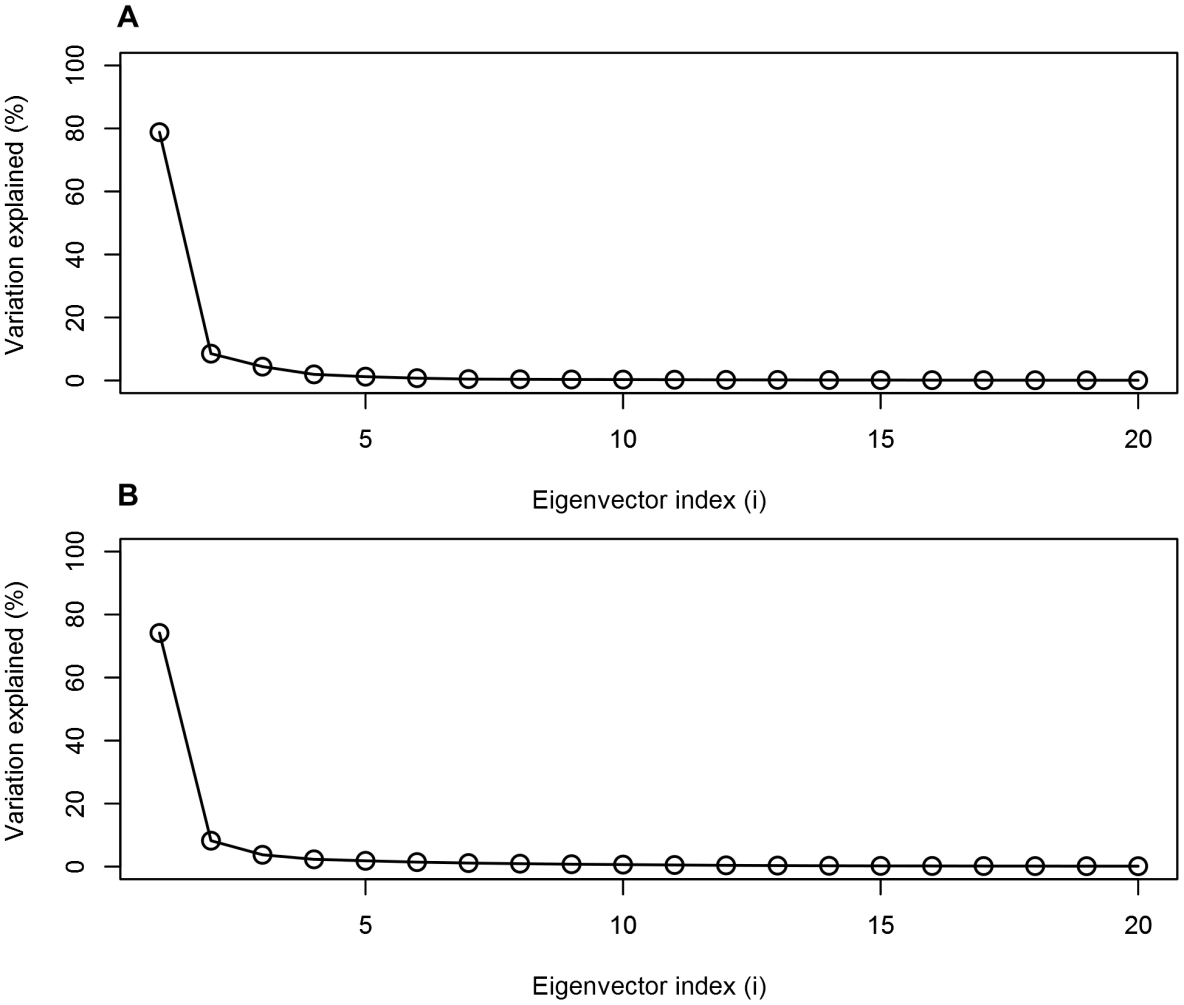
Contribution of the first twenty PCA eigenvectors to the overall closing motion of the PSI. The contribution of the first 20 PCA eigenvectors to the closing motion of the PSI at active (**A**) and inactive (**B**) pH are presented. The first eigenvectors contribute 78.8% and 74.2% of the overall motions for the pH 4.5 and pH 7.4 simulations, respectively, and correspond to the collapse of helices α1/α2 onto helices α3/α4.

**Figure 7:**
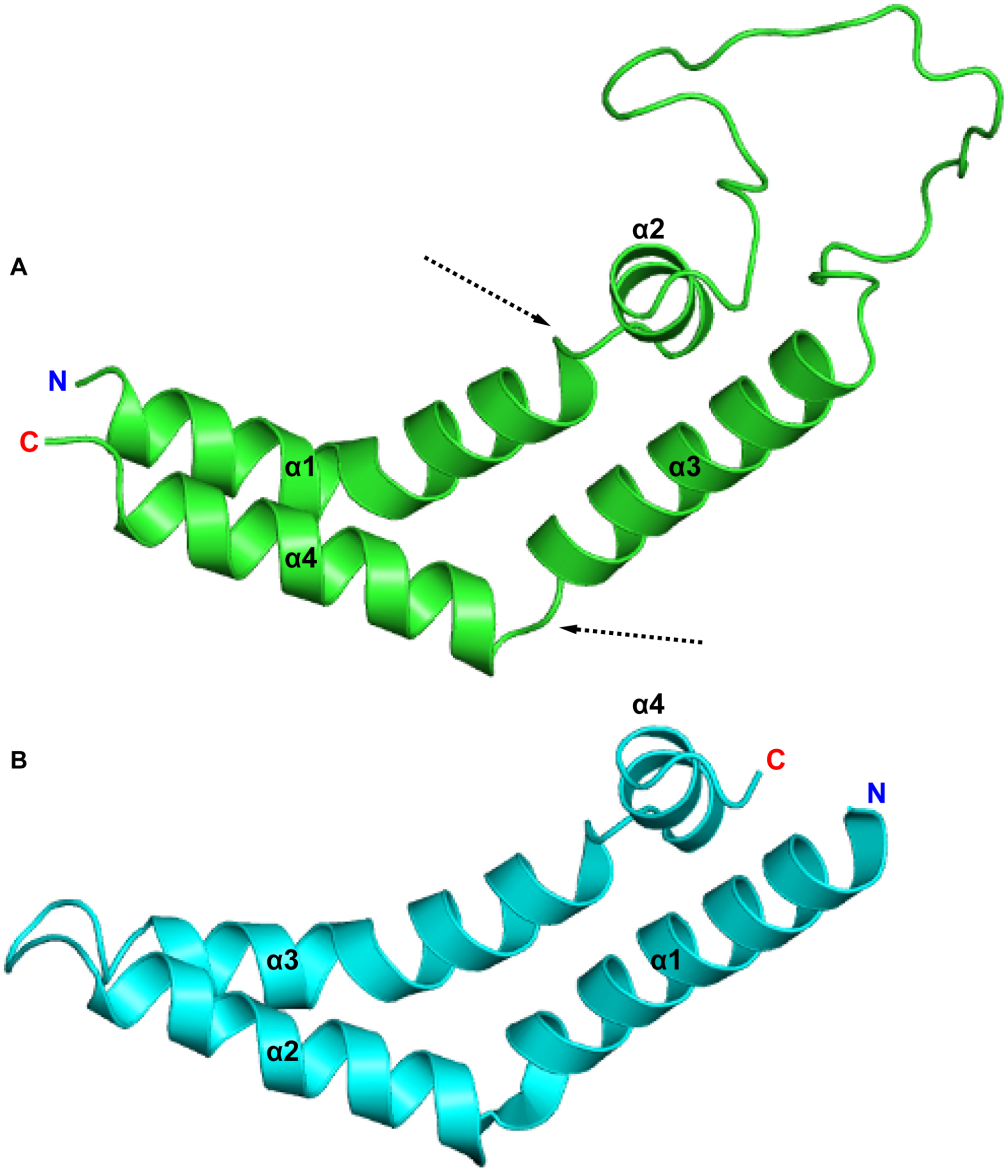
Structures of the PSI and orthorhombic Sap C. The crystal structure of potato (*Solanum tuberosum*) PSI (PDB ID 3RFI, **A**), with the missing linker region modelled and orthorhombic Sap C (PDB ID 2QYP, **B**) are presented. Like its Sap C homologue, potato PSI was crystalized as an extended dimer. The hinge-bending capability of the PSI is made possible by the flexible helix-helix junctions formed between α1/α2 and α3/α4, indicated by dashed arrows.

**Figure 8:**
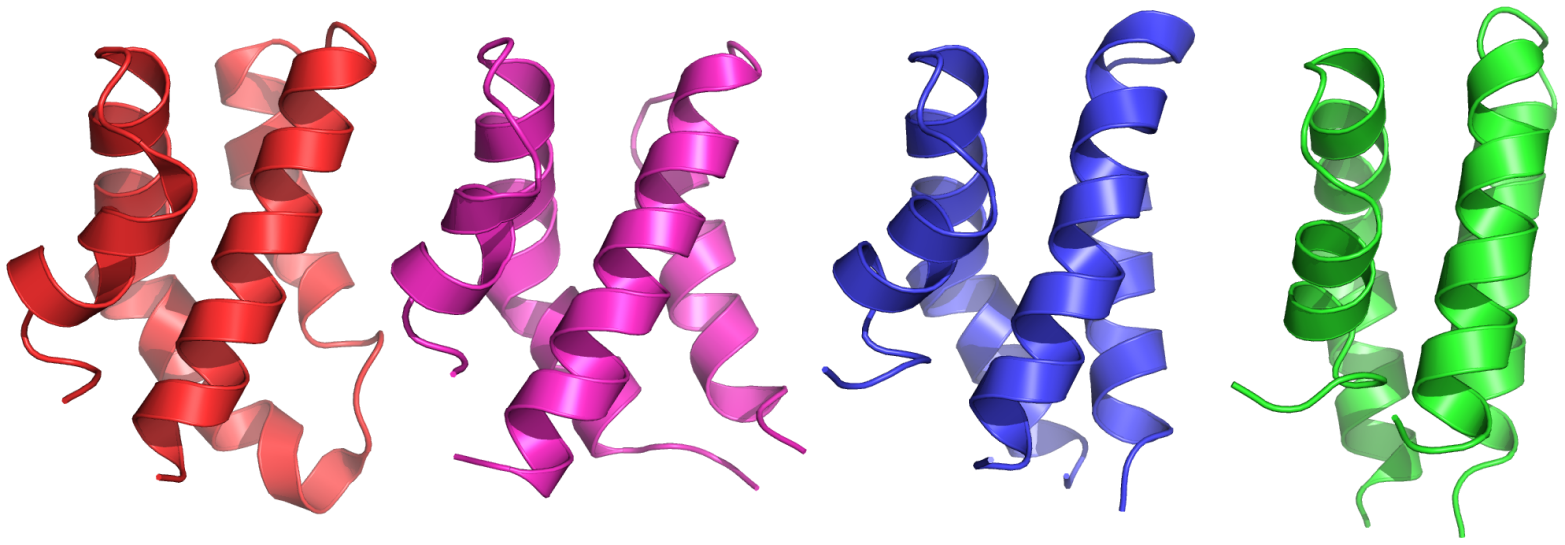
Comparison of folded potato PSI to other SAPLIPs. Structural comparison of the folded potato PSI at pH 4.5 (blue) and pH 7.4 (green), averaged over the last 200 ns of the simulation trajectories, to the crystal structure of barley PSI (PDB ID 1QDM, magenta) and the crystal structure Sap C (PDB ID 2GTG, red). Potato PSI simulated at pH 7.4, simulated with parameters closely resembling the experimental parameters used for both barley PSI and Sap C, exhibited a compact globular structure consisting of a distorted four-α-helix bundle characteristic of other SAPLIPs. Potato PSI simulated at pH 4.5 adopted a compact four-α-helix bundle structure not previously observed for any SAPLIP. The linker regions of potato PSI are omitted for clarity.

Principal component analysis (PCA) is a robust tool for identifying and separating the large-scale, and usually slowest, collective motions of atoms to reveal the largest contributors to atomic fluctuation of protein structures from the fast random internal motions [43], [44]. To examine tertiary structure dynamics of monomeric PSI, and assess potential influence of pH on protein folding, unrestrained MD simulations were performed on the extended PSI monomer in solution at both active and inactive pH values. PCA was then applied to the unrestrained MD simulations and conformational changes were examined. Monomer conformational stability was evaluated by calculating backbone RMSD after least-square fitting by superposing MD trajectories onto the PSI crystal structure. Simulations at both active and inactive pH produced similar trends in the evolution of RMSD, remaining stable with fluctuations in RMSD by approximately 0.2 nm –0.8 nm until approximately 230 ns (pH 4.5) and 198 ns (pH 7.4), suggesting that the PSI deviated little from the crystal structure. At these times, a transition in tertiary structure occurred in which the RMSD brusquely increased 1.2 nm –1.4 nm after which the RMSD remained stable upon adopting a new conformation (Figure 5). The extended conformation closed in on itself and adopted the closed saposin fold characteristic of other SAPLIPs [13], [14], [18]–[25] irrespective of pH. Hence, the simulations essentially described a spontaneous tertiary structure transition from the open to closed state. As one might expect, the1D mode described for the first PC in either simulation corresponded to the closing motion of the PSI, accounting for approximately 78.8% and 74.2% of the overall motions for simulations at active and inactive pH, respectively (Figure 6). This closing motion corresponded to helices α1/α4 collapsing onto helices α2/α3, hinging at the flexible helix-helix junctions formed between α1/α2 and α3/α4 (Figure 7). At pH 7.4, the second PC was characterized by a slight twisting motion of the terminal helices (α1 and α4) relative to helices α2/α3 and was responsible for the characteristic distortion of the α-helix bundle typical for the saposin fold (Figure 8), a phenomenon that was not observed in the active pH simulation. Subsequent PCs showed diminished contributions of conformational changes to overall motions of the PSI.

**Figure 9:**
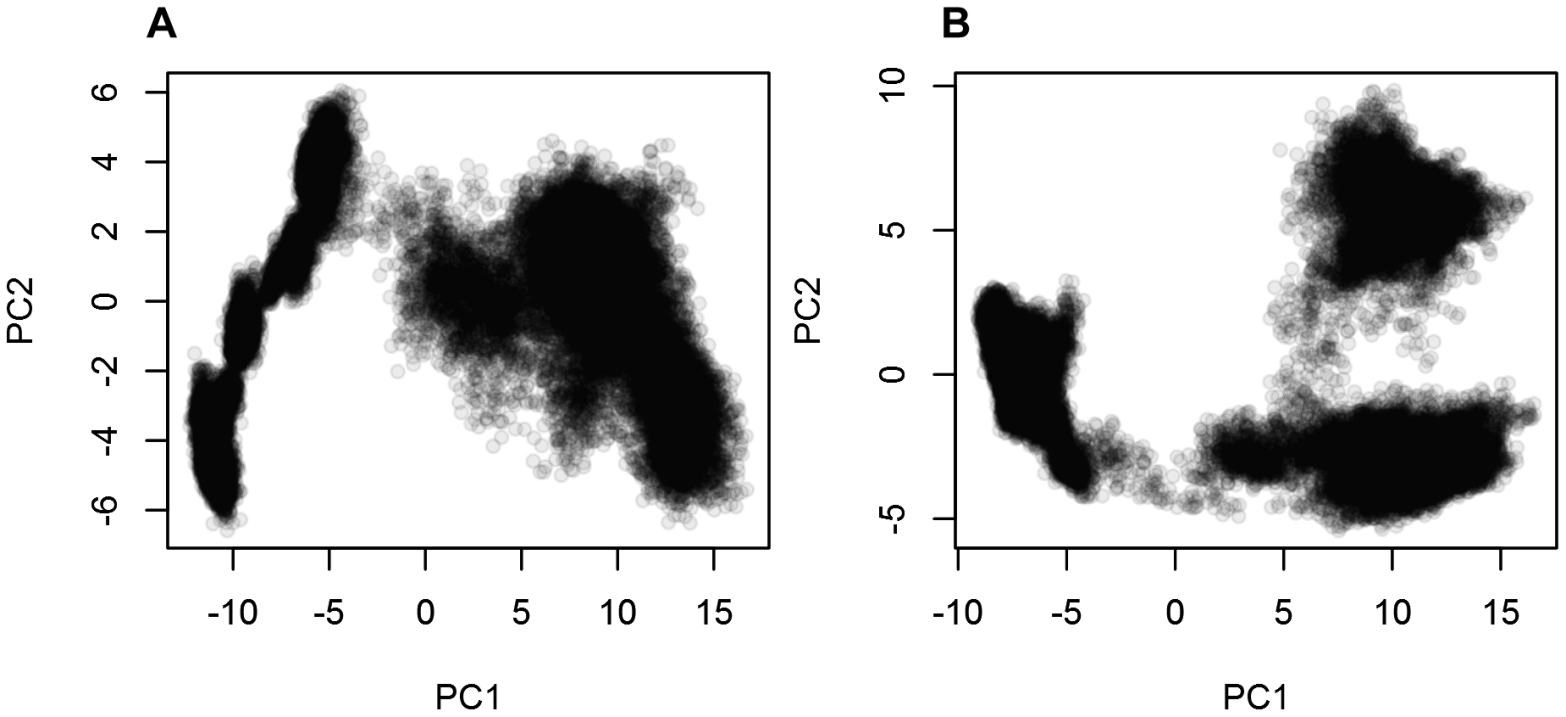
Two-dimensional projections of the first two eigenvectors of the PSI monomer. Projection of the first two eigenvectors of the unbiased PSI simulations at pH 4.5 (**A**) and pH 7.4 (**B**). Both simulations transited from the extended dimer-like structure to a saposin fold-like conformation over the course of the 500 ns trajectories. The inactive pH simulation transited through three distinct clusters whereas the active pH simulation transitioned through several microstates before becoming trapped in the last densely populated cluster. The differences in the essential subspace sampled by the two differing pH ranges may be due to unspecific (hydrophobic) interactions sampled in the pH 4.5 simulations where charge neutralization minimizes like-charge repulsions.

Two-dimensional projections of the active and inactive trajectories onto their respective first and second PCs showed that the PSI explores a wide range of conformational space (Figure 9). The conformer plot for the inactive simulation (Figure 9B) revealed that the PSI transits through three distinct conformational states corresponding to three distinct minima, after which it becomes trapped in a third and final state. The first conformational state corresponds to the extended open crystal structure. After sampling the essential subspace near the starting conformation, the monomer then transitions to a second, discreet state as the protein begins to jackknife. This intermediate conformation corresponds to a quasi-folded tertiary structure in which helices α1 and α4 begin to collapse onto helices α2 and α3, thus forming the beginnings of the characteristic 4-helix bundle observed for all known SAPLIPs [8]. The third and final cluster is the most densely populated and closely packed cluster corresponding to a distorted helix bundle tertiary structure like that of the characteristic saposin-fold. Similarly, the active pH simulation showed a transition from the initial open extended structure to a quasi-folded, compact 4-helix bundle tertiary structure (Figure 9A). However, unique to the active pH simulation were several microstates sampled along the second PC before finally becoming trapped in the densely populated final cluster.

### Radius of gyration measurements for investigating PSI compaction

**Figure 10:**
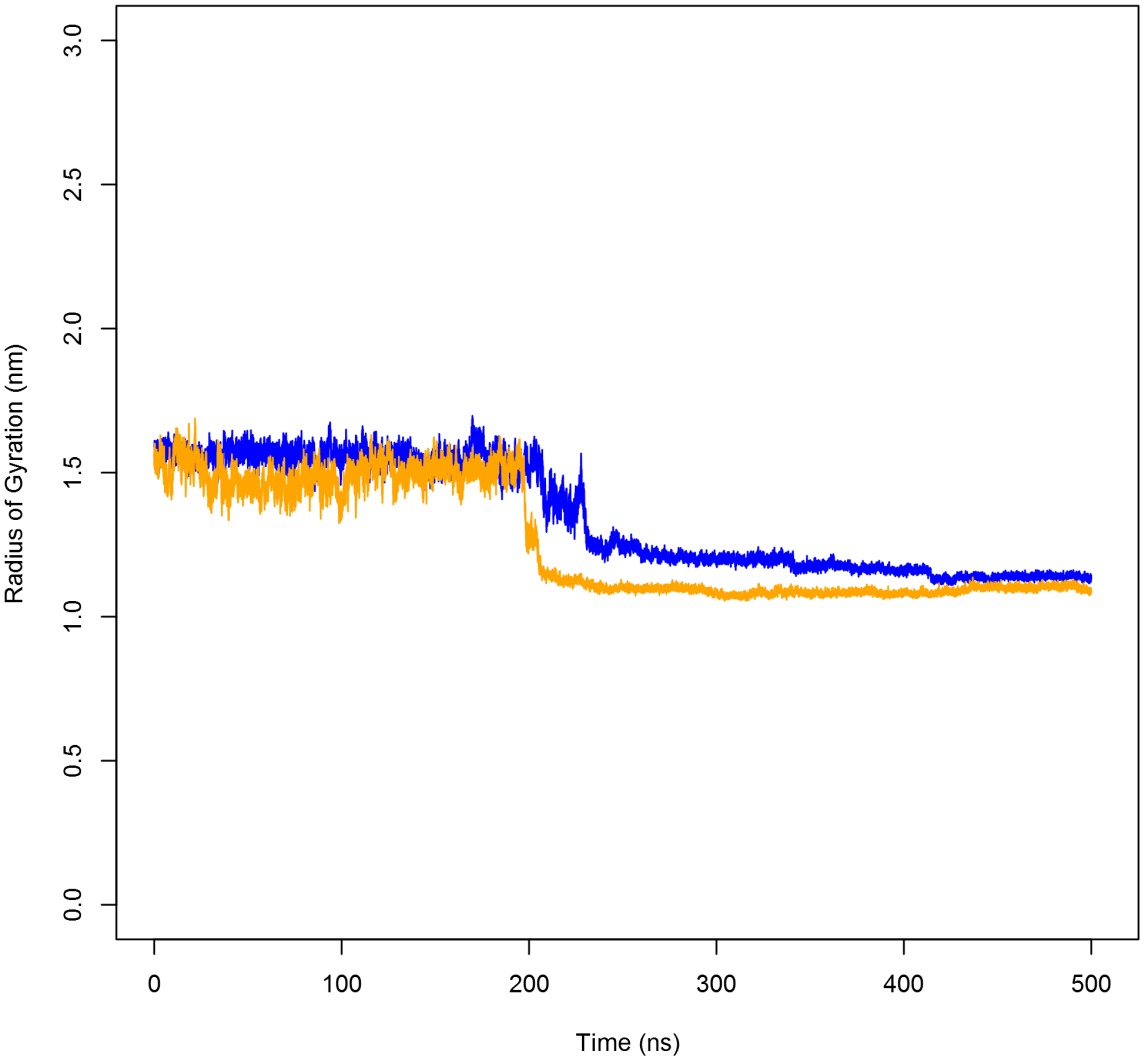
Radius of gyration (R_g_) of the PSI over the time course of the simulations. R_g_ of the PSI at pH 4.5 (blue line) and pH 7.4 (orange line) as a function of time. In either case, the PSI was free to move in the extended state. Upon adoption of a saposin-like fold, the collapse of the hydrophobic concave face of the PSI onto itself limits movement, thereby restricting water access to the hydrophobic core.

To monitor compaction of the PSI monomer as indicated from the principal component analyses, the radius of gyration (R_g_) was determined for hydrophobic residues located in helical regions (Figure 10). For both the active and inactive pH simulations, initial R_g_ corresponded to fluctuations in the PSI open conformation. At approximately 200 ns, a sharp decrease in R_g_ occurred corresponding to folding events related to the hydrophobic collapse of the concave face in which the stem formed between the N- and C-termini folded over onto helices α2/α3. This process corresponded to the large, abrupt changes in RMSD as well as the first PC observed at this time. Post-collapse, the lowered R_g_ values and the scarcity in R_g_ deviation throughout the remainder of the simulations were consistent with the adoption of a stable tertiary structure.

The adoption of the saposin fold-like tertiary structure for monomeric PSI is made possible by the hinges formed at the flexible helix-helix junctions between α1/α2 and α3/α4. Similar to the orthorhombic saposin crystal structure (PDB ID 2QYP) [22], extended PSI had obtuse opening angles of 109° and 110° for the active and inactive pH simulations, respectively, as measured at the Cα atoms of Pro66, Glu85 and Lys101 in which the hinge between helices α3 and α4 open about Glu85 (Figure 7A). Upon closing, the opening angles closed to approximately 23° and 33° for the pH 4.5 and 7.4 simulations, respectively, in agreement with the 34° opening angle measured at the Cα atoms of Pro67, Asn86 and Arg102 of the resolved portion of the closed HvAP PSI crystal structure [25]. Some α-helical secondary structure was lost at the helix-helix junctions during folding, transitioning to random coil to accommodate the movement of side chains towards the hydrophobic concave face of the PSI. The adoption of a saposin-like fold is further reinforced by the low RMSD between the resolved 4-helical bundle of the HvAP PSI and closed StAP PSI structures generated through MD (1.060 Å and 0.6477 Å for pH 4.5 and pH 7.4, respectively). All data are available upon request.

## Discussion

### Stability and dissociation of the PSI dimer

The calculated PMFs describing the dissociation of the PSI dimer at pH 3.0 and pH 7.4 gave Δ*G*_dissociation_ values of 108.8 kJ mol^−1^ and 95.7 kJ mol^−1^, respectively. As expected, the large free energy requirement can be attributed to the need to sequester the hydrophobic concave face of the PSI from solution, thereby minimizing entropy associated with the exposure of hydrophobic residues. Though both dimers form stable conformations, it should be noted that greater binding between the monomers is achieved at acidic pH, and the major reason for this is likely charge neutralization of carboxylic acid groups on glutamic and aspartic acid residues. It would be expected that electrostatic repulsion between monomers would thus be lowered allowing hydrophobic interactions at the dimer interface to dominate. An analogous phenomenon is seen with membrane-bound Sap C where neutralizing its negatively charged electrostatic surface removes membrane-protein charge-charge repulsion [19] thereby mitigating the unfavourable introduction of charges into the bilayer apolar hydrophobic environment. Furthermore, the calculated RMSD, RMSF and minimum distance maintained between the two monomers (see Figures 1–3) were consistent with the stability gained by folding the two monomers into a compact globular quaternary structure, limiting potential changes in tertiary structure.

Maintaining hydrophobic contact between the residues lining the concave face of the PSI is the driving force for preserving the quaternary structure and stability of the overall dimer, which may be related to the relative stability of the dimer at acidic pH relative to that at neutral pH. As the dimer experiences a lower free energy barrier to dissociation at neutral pH, the dimer quaternary structure can be interpreted as being less stable leading to eventual dissociation.

The larger free energy requirement for dissociation at acidic pH may be indicative of a physiological necessity. It has been established *a priori* that the PSI is active against bilayers at acidic pH [11], [25], [27], [45]. We hypothesise that the dimer formation at acidic pH may represent a particular functional quaternary structure. Baoukina and Tieleman [46], [47] previously concluded that covalently linked antiparallel lung surfactant protein B (SP-B) dimers, analogous to StAP PSI dimer, mediate faster kinetics of monolayer folding. SP-B dimers promoted bilayer folding and eventual formation of hemifusion-like stalk connections similar to those observed in vesicle fusion [46], [47]. It is theorised in the present study that PSI dimers may also function in a similar manner. Such a pH-dependence for a quaternary structure-function relationship is further supported by the observed StAP PSI capacity to induce bilayer fusion of large unilamellar vesicles (LUV) at acidic pH, causing both membrane disruption and fusion [11], and is supported by previous research examining the roles of the PSI in vesicle disruption and membrane targeting [26], [27], [45]. The idea that the dimer serves a functional role in bilayer disruption and fusion is also consistent with the “clip-on” model for Sap C-mediated vesicle fusion, proposed by Wang *et al*. [10] and further appended by Rossmann *et al*. [22]. This model hypothesises that Sap C dimers can bind to two vesicles, interacting with the membrane in a similar fashion to Sap C monomers through domain swapping, and thereby bring adjacent bilayers close enough to mediate fusion. Considering the similarities in structure and dimer stability pH-dependence, it stands to reason that our proposed model suggests a possible commonality between the Sap C “clip-on” model and the PSI mode of membrane interaction.

### Conformational flexibility and adoption of the saposin-fold

Insight into conformational changes can be gained by projecting the MD trajectory onto the subspace spanned by the two largest (typically the first and second) principal components [43], [48]. In doing so, it is possible to characterize the transitions from the open, extended conformation of the PSI monomer to the closed saposin fold-like structure seen in the unrestrained simulations. As well, any possible intermediate structures that may be adopted during the opening-to-closing transition can be observed, providing a map of the overall structural variability of the PSI. The resultant conformer plots thus provide the means to interpret the conformational changes sampled by the unrestrained MD simulations and express the relationships between these conformers. Unrestrained MD simulations of the extended PSI monomer suggested that the PSI adopts a closed saposin-like conformation independent of pH.

Principal component analysis performed on the MD trajectories revealed that the first PC corresponds to the closing of the PSI, accounting for 78.8% and 74.2% of the overall motion of the protein for the active and inactive pH simulations, respectively. Analogous to the PSI dimer, it is postulated that the closing motion observed in monomeric PSI arises from the need to reduce the entropy gained from exposure of these hydrophobic residues to water. Two-dimensional projections of the first two PCs revealed that the PSI transitions from an extended state to one or more intermediates before finally closing in on itself. Although the 2D projections sampled similar conformational space, the conformer plots of the active and inactive pH simulations differed in that the active pH simulation sampled several microstates before settling into a minimum and adopting a saposin-like closed motif. This differed from the inactive pH simulation in which the PSI sampled only three distinct states corresponding to energy minima for the initial structure, a molten globular structure, and finally a closed saposin-like tertiary structure. It is at this state that the concave face of the PSI has formed a hydrophobic core at its centre. These differences may be attributed to the differing electrostatic makeup of the two systems; negative charges on Glu and Asp are at least partially neutralized at active pH resulting in an overall positively charged protein, whereas both negative and positive residues exist in the neutral pH simulations allowing for potential intra-peptide salt bridging or potentially different hydrogen bonding patterns.

The conformational changes adopted by the PSI as it transits from extended to closed conformation were attributed to the high degree of conformational flexibility at the hinge-bending regions of the helix-helix junctions, similar to what is observed in other SAPLIPs. The latter is a common characteristic for saposin members of the SAPLIP family which have been shown to have the capacity to exist both in substrate-free closed and in extended lipid- or peptide-bound conformations [22]–[24]. For the PSI, this flexibility is made possible in part by the local dynamics of side chains. Hydrophobic residues located in the helical regions orient themselves such that their side chains are involved in the formation of the tight dimer interface (as observed in the unrestrained dimer simulations), induced by the presence of inter-protein hydrophobic interactions. The helix orientations in this packing motif are mimicked by monomeric PSI as it closes in a domain-swapped fashion in that helices α3 and α4 twist about their helical axes thereby maximizing intra-protein hydrophobic contacts. This folding process is marked by the hydrophobic regions of the four helices collapsing on themselves thereby minimizing contact with the polar environment and concomitantly maximizing aqueous contact with the polar outer surfaces.

The dynamics of PSI closure suggest two possible structures for the PSI, and that pH influences these conformational differences. To date, the only SAPLIP pH-structure report has been for Sap C in which a reduction in pH from 6.8 to 5.4 did not result in observable conformational changes [23]. It should be noted, however, that Sap C acidification occurred with monomeric protein already folded to a local minimum having adopted the characteristic saposin fold. This is in contrast to the present study in which the open PSI structure was allowed to explore a large degree of conformational space as it closed to a local energy minimum. The similar structure for the neutral StAP PSI ensemble in the present study, and that for HvAP PSI [25], as well as the low RMSDs, may indicate that the classical saposin-fold is pH-dependant for at least some SAPLIP cases. The neutral pH StAP PSI simulation and HvAP crystallography [25] used similar experimental parameters (i.e., 100 mM NaCl and neutral pH) suggesting that the acidic pH saposin-like fold observed in the present study likely presents a derivative of the classic saposin fold and it would be expected to be adopted by other SAPLIPs having similar structures and pH-function dependencies.

The present study undertook a comprehensive analysis of the PSI to identify conformational changes due to differences in pH and to assess the potential impact that these changes may have on protein function. Free energy changes for PSI dimer dissociation at acidic and neutral pH were predicted by steered MD simulations in combination with umbrella sampling. These identified key differences in binding affinities indicating that the PSI has a preference for maintaining the dimer quaternary structure at acidic (active) pH due to the higher free energy requirement for dissociation. In conclusion, we postulate that the preference for dimerization may be indicative of a functional structure that plays a role in membrane binding and vesicle fusion. PCA of unrestrained MD simulations of the PSI monomer after separation from the dimer complex was then used to assess conformational changes adopted by the monomers. Although monomeric PSI folded to a closed conformation regardless of pH, the final closed structures differed in that the pH 7.4 PSI adopted a tertiary structure consistent with the characteristic saposin-fold whereas a distinct saposin-like fold was observed at pH 4.5. This acidified PSI structure presents the first example of an alternative saposin-fold motif for any member of the large and diverse SAPLIP family.

## Methods

### Initial models

In the present study, the high resolution (1.9 Å) X-ray crystal structure of extended potato (*Solanum tuberosum*) PSI (PDB ID 3RFI) [11] was used as the template structure for SMD simulations. Chain A was used for the unrestrained MD simulations of the PSI monomer. The linker region (residues 40–63) connecting helices α1/α2 to α3/α4 was not resolved in the original crystal structure. As such, MODELLER 9v8 was used to build the missing linker region *ab initio* and modeled as random coil [49], [50]. Hydrogen atoms were added for all titratable residues in accordance to their calculated protonation states as determined using the H++ web server [51]–[53] using an internal protein dielectric constant and solvent dielectric constant of 10 and 80, respectively, with sodium chloride added at 140 mM or 100 mM for the SMD dimer dissociation (pH 3.0 and 7.4) and unrestrained MD monomer (pH 4.5 and 7.4) simulations, respectively.

### Unrestrained molecular dynamics system setup

All simulations and analyses were carried out using the GROMACS software suite, Version 4.5.5 [54]–[56] employing the Amber99sbnmr1-ILDN force field [57]–[59]. For each simulation, periodic boundary conditions were applied in all dimensions. The PSI was centred in a cubic box such that the protein was positioned at least 1.2 nm from the box edge and hydrated using the TIP3P explicit water model [60] to solvate the system. Sodium and chloride counterions were added at 140 mM or 100 mM concentrations for the SMD and unrestrained MD simulations, respectively, to produce electroneutral systems. Short-range electrostatic interactions were cut off at 8 Å whilst long-range electrostatic interactions were calculated using the particle-mesh Ewald (PME) summation method [61] with fourth order B-spine interpolation and a maximum grid spacing of 1.2 Å. A twin-range van der Waals cut-off was employed (0.8/1.0 nm) and an integration time step of 2 fs was used with neighbour searching performed every 5 steps with all bond lengths being constrained using the linear constraint solver (LINCS) algorithm [62].

Each simulation was prepared in 3 phases before production runs were performed. In the first phase, the protein was energy minimized using the steepest decent algorithm with position restraints placed on all heavy atoms (*k*_PR_ = 1000 kJ mol^−1^ nm^−2^) until the maximum force converged to ≤500 kJ mol^−1^ nm^−1^. In the next phase, the system was equilibrated for 1 ns with position restraints placed on all heavy atoms in the canonical ensemble using the Berendsen weak coupling method [63] with temperature maintained at 303.15 K (τ_T_ = 0.1 ps). This equilibration was followed by another 1 ns position-restrained simulation in the isobaric-isochoric ensemble. Again, the Berendsen weak coupling method was used to maintain temperature at 303.15 K and pressure isotropically coupled at 1 bar (τ_P_ = 1.0 ps). The isothermal compressibility of the system was set to 4.5×10^−5^ bar^−1^. For the production unrestrained MD simulations, position restraints were removed. The velocity rescale (v-rescale) algorithm [64] was used to maintain the temperature of the system at 303.15 K (τ_T_ = 0.1 ps) and the pressure was again maintained at 1 bar using the Parrinello-Rahman [65], [66] barostat (τ_P_ = 2.0 ps) in the isobaric-isochoric ensemble with long-range dispersion correction applied for both the energy and pressure terms. Production simulations were conducted for 500 ns.

### Steered molecular dynamics

**Figure 11:**
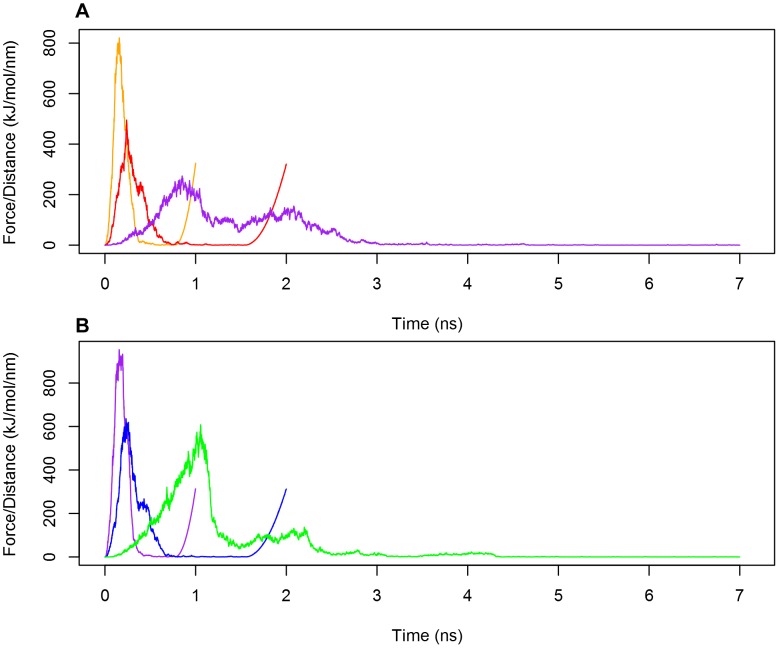
Force-Time curves of the dissociation of the PSI dimers using differing pull rates. Force-Ttime curves for pH 3.0 (**A**) and pH 7.4 (**B**) pulling simulations using 10 nm ns^−1^ (orange line in **A**, purple line in **B**), 5 nm ns^−1^ (red line in **A**, blue line in **B**) and 1 nm ns^−1^ (purple line in **A**, green line in **B**). The curves exhibit similar shapes overall suggesting that pull rate does not appreciably affect dimer dissociation.

Equilibrated starting structures of the PSI dimer for the pH 3.0 and pH 7.4 SMD simulations were generated following the same procedure used for the unrestrained MD simulations with production MD conducted for 100 ns. The resultant structures were then used as the starting configuration for the corresponding SMD pulling simulations. The PSI dimer was placed in a rectangular box large enough to accommodate separation of the dimer along the *z* axis whilst satisfying the minimum image convention. The dimer was then subjected to energy minimization and equilibration in both the canonical and isobaric-isochoric ensemble again as described above. For the SMD pulling simulations, position restraints were removed from chain B of the PSI dimer while heavy atoms of chain A were harmonically restrained (*k*_PR_ = 1000 kJ mol^−1^ nm^−2^) in a similar fashion to that used in the equilibration phases. Chain A was used as an immobile reference for chain B pulling. The 1D reaction coordinate was chosen to be the distance along the *z* axis between the COMs of the two PSI monomers. Chain B was pulled away from chain A along the *z* axis for 1 ns with a constant velocity of 10 nm ns^−1^ using an elastic spring (*k* = 1000 kJ mol^−1^ nm^−2^) positioned at the COM of the peptide. Trajectories at slower pulling rates (5 ns nm^−1^ and 1 ns nm^−1^) were also tested to assess the influence of pulling forces on the structure as force is applied. These slower pulling rates resulted in similar force-time curves and similar overall trajectories (Figure 11) [67]. As such, the faster pulling rate was used for experimental SMD simulations to minimize usage of computational resources.

### Umbrella sampling and determination of PMF

The pH 3.0 and pH 7.4 trajectories from SMD pulling simulations were used to generate sampling windows along the reaction coordinate. Windows were spaced between 0.5–2.0 Å for the first 2.5 nm followed by approximately 2.0 Å spacing until the overall distance between the COMs between chains A and B was approximately 6.0 nm. This resulted in 43 and 45 sampling windows being selected for the pH 3.0 and pH 7.4 simulations, respectively. MD was conducted for each window for 15 ns with a harmonic restraint (*k*_PR_ = 1000 kJ mol^−1^ nm^−2^) applied to chain B to fix the peptide along the reaction coordinate and then the PMF was constructed. The unbiased PMF was calculated using WHAM and the Δ*G*_dissociation_ was evaluated as the difference in energy between the plateau and energy minimum along the PMF curve [68].

### Principal component analysis

PCA was used to identify principal modes of motion sampled during the unrestrained MD simulations of the extended PSI monomer at pH 4.5 and pH 7.4. A covariance matrix of the backbone atoms in the monomer was constructed using the PSI trajectories. The matrices were then diagonalized yielding eigenvectors and their corresponding eigenvalues revealing both the directions and amplitudes of motion, respectively. Projection of the two largest eigenvectors onto 2D space was then used to quantitatively compare the ability of each ensemble to sample varying regions of conformational space.

## References

[1] Davies DR (1990) The structure and function of the aspartic proteinases. Annu Rev Biophys Biophys Chem 19: 189–215. 2194475 10.1146/annurev.bb.19.060190.001201

[2] Rawlings ND, Barrett AJ, Bateman A (2010) MEROPS: The peptidase database. Nucleic Acids Res 38: D227–D233. 19892822 10.1093/nar/gkp971 PMC2808883

[3] Runeberg-Roos P, Törmäkangas K, Östman A (1991) Primary structure of a barley-grain aspartic proteinase. Eur J Biochem 202: 1021–1027. 1722454 10.1111/j.1432-1033.1991.tb16465.x

[4] Cordeiro MC, Xue Z, Pietrzak M, Salomé Pais M, Brodelius PE (1994) Isolation and characterization of a cDNA from flowers of *Cynara cardunculus* encoding cyprosin (an aspartic proteinase) and its use to study the organ-specific expression of cyprosin. Plant Mol Biol 24: 733–741. 8193298 10.1007/BF00029855

[5] Simões I, Faro C (2004) Structure and function of plant aspartic proteinases. Eur J Biochem 271: 2067–2075. 15153096 10.1111/j.1432-1033.2004.04136.x

[6] Törmäkangas K, Kervinen J, Östman A, Teeri T (1994) Tissue-specific localization of aspartic proteinase in developing and germinating barley grains. Planta 195: 116–125.

[7] Guruprasad K, Törmäkangas K, Kervinen J, Blundell TL (1994) Comparative modelling of barley-grain aspartic proteinase: A structural rationale for observed hydrolytic specificity. FEBS Lett 352: 131–136. 7925961 10.1016/0014-5793(94)00935-x

[8] Bruhn H (2005) A short guided tour through functional and structural features of saposin-like proteins. Biochem J 389: 249–257. 15992358 10.1042/BJ20050051 PMC1175101

[9] Vaccaro AM, Tatti M, Ciaffoni F, Salvioli R, Serafino A, et al (1994) Saposin C induces pH-dependent destabilization and fusion of phosphatidylserine-containing vesicles. FEBS Lett 349: 181–186. 8050562 10.1016/0014-5793(94)00659-8

[10] Wang Y, Grabowski GA, Qi X (2003) Phospholipid vesicle fusion induced by saposin C. Arch Biochem Biophys. 415: 43–53. 10.1016/s0003-9861(03)00219-4 12801511

[11] Bryksa BC, Bhaumik P, Magracheva E, De Moura DC, Kurylowicz M, et al (2011) Structure and mechanism of the saposin-like domain of a plant aspartic protease. J Biol Chem 286: 28265–28275. 21676875 10.1074/jbc.M111.252619 PMC3151071

[12] Matsuda J, Vanier MT, Saito Y, Tohyama J, Suzuki K, et al (2001) A mutation in the saposin A domain of the sphingolipid activator protein (prosaposin) gene results in a late-onset, chronic form of globoid cell leukodystrophy in the mouse. Hum Mol Genet 10: 1191–1199. 11371512 10.1093/hmg/10.11.1191

[13] Willis C, Wang CK, Osman A, Simon A, Pickering D, et al (2011) Insights into the membrane interactions of the saposin-like proteins *Na*-SLP-1 and *Ac*-SLP-1 from human and dog hookworm. PLoS ONE 6: e25369. 21991310 10.1371/journal.pone.0025369 PMC3184995

[14] Liepinsh E, Andersson M, Ruysschaert J, Otting G (1997) Saposin fold revealed by the NMR structure of NK-lysin. Nat Struct Mol Biol 4: 793–795. 10.1038/nsb1097-793 9334742

[15] Anderson DH, Sawaya MR, Cascio D, Ernst W, Modlin R, et al (2003) Granulysin crystal structure and a structure-derived lytic mechanism. J Mol Biol 325: 355–365. 12488100 10.1016/s0022-2836(02)01234-2

[16] Guevara MG, Veríssimo P, Pires E, Faro C, Daleo DR (2004) Potato aspartic proteases: induction, antimicrobial activity and substrate specificty. J Plant Pathol 86: 233–238.

[17] Mendieta JR, Fimognari C, Daleo GR, Hrelia P, Guevara MG (2010) Cytotoxic effect of potato aspartic proteases (StAPs) on Jurkat T cells. Fitoterapia 5: 329–335. 10.1016/j.fitote.2009.10.004 19825400

[18] Jongstra J, Schall TJ, Dyer BJ, Clayberger C, Jorgensen J, et al (1987) The isolation and sequence of a novel gene from a human functional T cell line. J Exp Med 165: 601–614. 2434598 10.1084/jem.165.3.601 PMC2188281

[19] de Alba E, Weiler S, Tjandra N (2003) Solution structure of human saposin C: pH-dependent interaction with phospholipid vesicles. Biochemistry 42: 14729–14740. 14674747 10.1021/bi0301338

[20] Ahn VE, Faull KF, Whitelegge JP, Fluharty AL, Privé GG (2003) Crystal structure of saposin B reveals a dimeric shell for lipid binding. Proc Natl Acad Sci U S A 100: 38–43. 12518053 10.1073/pnas.0136947100 PMC140876

[21] Ahn VE, Leyko P, Alattia J, Chen L, Privé GG (2006) Crystal structures of saposins A and C. Protein Sci. 15: 1849–1857. 10.1110/ps.062256606 PMC2242594 16823039

[22] Rossmann M, Schultz-Heienbrok R, Behlke J, Remmel N, Alings C, et al (2008) Crystal structures of human saposins C and D: Implications for lipid recognition and membrane interactions. Structure 16: 809–817. 18462685 10.1016/j.str.2008.02.016

[23] Hawkins CA, Alba Ed, Tjandra N (2005) Solution structure of human saposin C in a detergent environment. J Mol Biol 346: 1381–1392. 15713488 10.1016/j.jmb.2004.12.045

[24] Popovic K, Holyoake J, Pomès R, Privé GG (2012) Structure of saposin A lipoprotein discs. Proc Natl Acad Sci U S A 109: 2908–2912. 22308394 10.1073/pnas.1115743109 PMC3286916

[25] Kervinen J, Tobin GJ, Costa J, Waugh DS, Wlodawer A, et al (1999) Crystal structure of plant aspartic proteinase prophytepsin: Inactivation and vacuolar targeting. EMBO J 18: 3947–3955. 10406799 10.1093/emboj/18.14.3947 PMC1171470

[26] Törmäkangas K, Hadlington JL, Pimpl P, Hillmer S, Brandizzi F, et al (2001) A vacuolar sorting domain may also influence the way in which proteins leave the endoplasmic reticulum. Plant Cell 13: 2021–2032. 11549761 10.1105/TPC.000533 PMC139449

[27] Egas C, Lavoura N, Resende R, Brito RMM, Pires E, et al (2000) The saposin-like domain of the plant aspartic proteinase precursor is a potent inducer of vesicle leakage. J Biol Chem 275: 38190–38196. 10982803 10.1074/jbc.M006093200

[28] Binnig G, Quate CF, Gerber C (1986) Atomic force microscope. Phys Rev Lett 56: 930–933. 10033323 10.1103/PhysRevLett.56.930

[29] Ashkin A, Dziedzic JM, Bjorkholm JE, Chu S (1986) Observation of a single-beam gradient force optical trap for dielectric particles. Opt Lett 11: 288–290. 19730608 10.1364/ol.11.000288

[30] Izrailev S, Stepaniants S, Isralewitz B, Kosztin B, Lu H, et al.. (1999) Steered molecular dynamics. In: Deuflhard P, Hermans J, Leimkuhler B, Mark A, Skeel RD, Reich S, editors. Computational Molecular Dynamics: Challenges, Methods, Ideas. Berlin: Springer-Verlag. 39–65.

[31] Sotomayor M, Schulten K (2007) Single-molecule experiments *in vitro* and *in silico* . Science 316: 1144–1148. 17525328 10.1126/science.1137591

[32] West DK, Brockwell DJ, Olmsted PD, Radford SE, Paci E (2006) Mechanical resistance of proteins explained using simple molecular models. Biophys J 90: 287–297. 16214858 10.1529/biophysj.105.071035 PMC1367027

[33] González A, Perez-Acle T, Pardo L, Deupi X (2011) Molecular basis of ligand dissociation in β-adrenergic receptors. PLoS ONE 6: e23815. 21915263 10.1371/journal.pone.0023815 PMC3168429

[34] Kalikka J, Akola J (2011) Steered molecular dynamics simulations of ligand-receptor interaction in lipocalins. Eur Biophys J 40: 181–194. 21072508 10.1007/s00249-010-0638-3

[35] Cuendet MA, Michielin O (2008) Protein-protein interaction investigated by steered molecular dynamics: The TCR-pMHC complex. Biophys J 95: 3575–3590. 18621828 10.1529/biophysj.108.131383 PMC2553100

[36] Torrie GM, Valleau JP (1977) Nonphysical sampling distributions in monte carlo free-energy estimation: Umbrella sampling. J Comput Phys 23: 187–199.

[37] Roux B (1995) The calculation of the potential of mean force using computer simulations. Comput Phys Commun 91: 275–282.

[38] NategholEslam M, Holland BW, Gray CG, Tomberli B (2011) Drift-oscillatory steering with the forward-reverse method for calculating the potential of mean force. Phys Rev E Stat Nonlin Soft Matter Phys 83: 021114. 21405825 10.1103/PhysRevE.83.021114

[39] Kosztin I, Barz B, Janosi L (2006) Calculating potentials of mean force and diffusion coefficients from nonequilibrium processes without Jarzynski’s equality. J Chem Phys 124: 064106. 10.1063/1.2166379 16483195

[40] Kästner J (2011) Umbrella sampling. Wiley Interdiscip Rev Comput Mol Sci 1: 932–942.

[41] Kumar S, Bouzida D, Swendsen RH, Kollman PA, Rosenberg JM (1992) The weighted histogram analysis method for free-energy calculations on biomolecules. I. The method. J Comput Chem 13: 1011–1021.

[42] Zhang BW, Brunetti L, Brooks CL (2011) Probing pH-dependent dissociation of HdeA dimers. J Am Chem Soc 133: 19393–19398. 22026371 10.1021/ja2060066 PMC3227773

[43] Amadei A, Linssen ABM, Berendsen HJC (1993) Essential dynamics of proteins. Proteins: Struct Funct Genet 17: 412–425. 8108382 10.1002/prot.340170408

[44] Berendsen HJ, Hayward S (2000) Collective protein dynamics in relation to function. Curr Opin Struct Biol 10: 165–169. 10753809 10.1016/s0959-440x(00)00061-0

[45] Frazão C, Bento I, Costa J, Soares CM, Veríssimo P, et al (1999) Crystal structure of cardosin A, a glycosylated and arg-gly-asp-containing aspartic proteinase from the flowers of *Cynara cardunculus* L. J Biol Chem. 274: 27694–27701. 10.1074/jbc.274.39.27694 10488111

[46] Baoukina S, Tieleman DP (2010) Direct simulation of protein-mediated vesicle fusion: Lung surfactant protein B. Biophys J. 99: 2134–2142. 10.1016/j.bpj.2010.07.049 PMC3042587 20923647

[47] Baoukina S, Tieleman D (2011) Lung surfactant protein SP-B promotes formation of bilayer reservoirs from monolayer and lipid transfer between the interface and subphase. Biophys J 100: 1678–1687. 21463581 10.1016/j.bpj.2011.02.019 PMC3072669

[48] Barrett CP, Hall BA, Noble MEM (2004) Dynamite: A simple way to gain insight into protein motions. Acta Crystallogr D Biol Crystallogr 60: 2280–2287. 15572782 10.1107/S0907444904019171

[49] Eswar N, Webb B, Marti-Renom MA, Madhusudhan MS, Eramian D, et al.. (2006) Comparative protein structure modeling using Modeller. Curr Protoc Bioinformatics Chapter 5: Unit 5.6. 10.1002/0471250953.bi0506s15 PMC4186674 18428767

[50] Fiser A, Do RK, Sali A (2000) Modeling of loops in protein structures. Protein Sci 9: 1753–1773. 11045621 10.1110/ps.9.9.1753 PMC2144714

[51] Gordon JC, Myers JB, Folta T, Shoja V, Heath LS, et al (2005) H++: A server for estimating pKas and adding missing hydrogens to macromolecules. Nucleic Acids Res 33: W368–W371. 15980491 10.1093/nar/gki464 PMC1160225

[52] Anandakrishnan R, Aguilar B, Onufriev AV (2012) H++3.0: Automating pK prediction and the preparation of biomolecular structures for atomistic molecular modeling and simulations. Nucleic Acids Res 40: W537–W541. 22570416 10.1093/nar/gks375 PMC3394296

[53] Myers J, Grothaus G, Narayanan S, Onufriev A (2006) A simple clustering algorithm can be accurate enough for use in calculations of pKs in macromolecules. Proteins 63: 928–938. 16493626 10.1002/prot.20922

[54] Van Der Spoel D, Lindahl E, Hess B, Groenhof G, Mark AE, et al (2005) GROMACS: Fast, flexible, and free. J Comput Chem 26: 1701–1718. 16211538 10.1002/jcc.20291

[55] Hess B, Kutzner C, van der Spoel D, Lindahl E (2008) GROMACS 4: Algorithms for highly efficient, load-balanced, and scalable molecular simulation. J Chem Theory Comput 4: 435–447. 26620784 10.1021/ct700301q

[56] Pronk S, Páll S, Schulz R, Larsson P, Bjelkmar P, et al (2013) GROMACS 4.5: A high-throughput and highly parallel open source molecular simulation toolkit. Bioinformatics 29: 845–854. 23407358 10.1093/bioinformatics/btt055 PMC3605599

[57] Lindorff-Larsen K, Piana S, Palmo K, Maragakis P, Klepeis JL, et al (2010) Improved side-chain torsion potentials for the amber ff99SB protein force field. Proteins 78: 1950–1958. 20408171 10.1002/prot.22711 PMC2970904

[58] Long D, Li DW, Walter KF, Griesinger C, Brüschweiler R (2011) Toward a predictive understanding of slow methyl group dynamics in proteins. Biophys J 101: 910–915. 21843482 10.1016/j.bpj.2011.06.053 PMC3175054

[59] Li DW, Brüschweiler R (2010) NMR-based protein potentials. Angew Chem Int Ed Engl 49: 6778–6780. 20715028 10.1002/anie.201001898

[60] Jorgensen WL, Chandrasekhar J, Madura JD, Impey RW, Klein ML (1983) Comparison of simple potential functions for simulating liquid water. J Chem Phys 79: 926–935.

[61] Darden T, York D, Pedersen L (1993) Particle mesh Ewald: An *N*·log(*N*) method for Ewald sums in large systems. J Chem Phys 98: 10089–10092.

[62] Hess B, Bekker H, Berendsen HJC, Fraaije JGEM (1997) LINCS: A linear constraint solver for molecular simulations. J Comput Chem 18: 1463–1472.

[63] Berendsen HJC, Postma JPM, van Gunsteren WF, DiNola A, Haak JR (1984) Molecular dynamics with coupling to an external bath. J Chem Phys 81: 3684–3690.

[64] Bussi G, Donadio D, Parrinello M (2007) Canonical sampling through velocity rescaling. J Chem Phys 126: 014101. 17212484 10.1063/1.2408420

[65] Parrinello M, Rahman A (1981) Polymorphic transitions in single crystals: A new molecular dynamics method. J Appl Phys 52: 7182–7190.

[66] Nosé S, Klein ML (1983) Constant pressure molecular dynamics for molecular systems. Mol Phys 50: 1055–1076.

[67] Lemkul JA, Bevan DR (2010) Assessing the stability of Alzheimer’s amyloid protofibrils using molecular dynamics. J Phys Chem B 114: 1652–1660. 20055378 10.1021/jp9110794

[68] Chen P, Kuyucak S (2011) Accurate determination of the binding free energy for KcsA-charybdotoxin complex from the potential of mean force calculations with restraints. Biophys J 100: 2466–2474. 21575581 10.1016/j.bpj.2011.03.052 PMC3093566

